# Progress in Surgery

**Published:** 1895-06-08

**Authors:** 


### PROGRESS IN SURGERY.

### THE SURGERY OF THE SPLEEN AND PANCREAS.

**Gun-shot Wound of the Spleen.**—Tiffany[1] reports the case of a negro, aged twenty years, who had been shot in the back. There was a bullet wound three inches to the left of the spine, just below the last rib. This wound was enlarged after the patient had been anæsthetised. The upper portion of the left kidney was found to be perforated, and dark blood flowed from the peritoneal cavity beyond. An incision was then made in the left linea semilunaris. A moderate amount of blood was free in the peritoneal cavity, and the spleen was found to be perforated about three inches from the lower free border. Unwilling to subject the patient to splenectomy, the author arrested the bleeding in the following manner: A long needle threaded with silk was passed entirely through the spleen central to and parallel with the bullet track; the long ligature was then tied over the free border of the organ, so as to press the surfaces of the wound together tightly enough to arrest bleeding, yet not to tear through the splenic tissue; the ends of the ligature were cut short, the peritoneal cavity cleansed by irrigation with hot water, and the abdominal wound closed. The kidney was tamponed with gauze through the dorsal wound. Convalescence was uneventful, and the patient left the hospital in about four weeks.

**Axial Rotation of the Spleen.**—A case of this rare condition is reported by Malins[2]. The left side of the abdomen was occupied by a large tumour. On abdominal section this was found to be the spleen enlarged, tense, and rotated for half a revolution upon its axis. The pedicle was nearly an inch in thickness. On attempting to restore the position, the twist resumed its abnormal state. Two thin, strong prepared silk ligatures were placed round the pedicle without transfixion, and the spleen was cut away. The patient made a good recovery. Subsequent examinations of the blood showed a diminution in the number of red blood corpuscles, and an increase in number of the white blood corpuscles. A similar case is reported by Conklin (*vide infra*) in a woman aged twenty-nine.

**Splenectomy.**—Ceci[3] reports two successful cases of splenectomy. The mortality for all cases is 51·6 per cent., but ranges in different types from 91·4 per cent. in cases of splenic hypertrophy with leukæmia, to 11·5 per cent. in essential idiopathic hypertrophy with ectopia. These percentages are deduced from a series of 145 reported splenectomies. Ceci's first case was that of a girl aged sixteen years, who had malarial fever at seven, and from that time the belly had slowly enlarged. The tumour was painful on movement. After operation the pallor was replaced by ruddy skin, there was slight swelling of the right tonsil, the thyroid continued normal, and there was no alteration of voice. Interrogated closely, she confessed to slight pains in the lower epiphysis of the femur, but the bones were of normal size and not tender. The second case was in a child aged thirteen years. Six months after operation she exhibited no trace of vicarious hypertrophy in other lymphatic tissues, and was in good general health. Successful cases of splenectomy are also recorded by Markoe[4] (two cases), Murphy[5], Czerny[6] (five cases), and Conklin[7]. The phenomena determined in man by the ablation of a diseased spleen have not as yet, says Vulpius[8], been sufficiently elucidated. In a small number of cases hypertrophy of the lymphatic glands has been observed; in some other cases goitre supervened, accompanied in one instance by the symptoms of Graves' disease. These changes, however, occurred so rarely that they cannot be considered as the direct consequence of the operation. Of greater importance are the cases in which careful examination of the blood has been made before and after operation. The manifestation most frequently observed was a more or less rapid increase of the leucocytes after ablation of the spleen. The question as to whether a part of the function of the spleen is to transform leucocytes into erythrocytes is a vexed one, but the conclusions arrived at are that ablation of the spleen is followed by a numerical decrease of the red, and increase of the white, blood corpuscles. With regard to compensatory hypertrophy of accessory spleens, it is not impossible that this compensation does take place, though it has not as yet been demonstrated after splenectomy in man. The presence of accessory spleens, for that matter, is extremely variable. Dupuytren states that they are especially found in the young, and especially in the fœtus. The existence or absence of these accessory glands may account in a certain measure for the differences observed in respect of changes in the blood, subsequently to splenectomy in man. Lastly, experiments on animals tend to show that, as far as hæmatopoiesis is concerned, supplementary functions may be assumed by the lymphatic glands and the marrow. Various experiments seem to show that the regeneration of the blood after hæmorrhages is somewhat slow in animals deprived of their spleen. On the other hand, Vulpius has not found that the consequences of splenectomy were more serious in animals which had before been subjected to blood-letting than in those from which no blood had previously been withdrawn.

**Inflammatory Affections of the Pancreas.**—In these cases, unless following injury, surgical treatment is seldom practised. Köste[9] has had under his observation four cases of suppuration and necrosis of the pancreas, and one case which did not go on to suppuration. Two of the cases operated on recovered, two died; the first case was diagnosed in the course of treatment, the succeeding ones were diagnosed. The patients were adults, aged from 22 to 48 years. The symptoms are as follows: Sudden severe illness usually in patient seemingly in good health, or after slight digestive derangement, vomiting, pain in the abdomen extending from the epigastrium, great prostration, usually constipation, abdominal distension and sense of pressure. The acute stage may very easily be mistaken for some other affection—gastro-duodenitis, poisoning, biliary colic, peritonitis, or intestinal occlusion. Sometimes the symptoms subside in a few days, then in one to three weeks recur. Death may occur in the acute stage from collapse; in the chronic the patient sinks slowly from fever, diarrhœa, and discharge of pus *per anum*. In some cases there is jaundice and bronzing of the skin. In the sub-acute cases a swelling appears in the epigastrium between the stomach and colon, extending to the left, and sometimes into the left lumbar region. Post-mortem observations and injection of colouring matter into the pancreas show that pus may pass in the following directions: (1) Distension and rupture into the bursa omentalis; (2) burrow down to the left side behind the descending colon, but sometimes going to the right side; (3) along the spinal column down between the layers of the mesocolon or mesentery. The diagnosis is based on the characteristic commencement of the affection, followed by the appearance of the epigastric tumour or swelling in the left lumbar region. The treatment is settled by the diagnosis; in abscess in the bursa omentalis, laparotomy, suture of the walls, drainage, a counter opening to be made if possible. In the retro-peritoneal form, incision in the lumbar region and freeing of the posterior peritoneal wall till the abscess is reached.

**Pancreatic Cyst.**—Zweifel[10] has successfully treated a case by extirpation in a patient aged 64, who had noticed a tumour under the costal arch for three years. The author discusses the treatment of pancreatic cysts by extirpation and drainage. Out of twelve cases treated by extirpation five died, whilst only two succumbed out of thirty-one treated by drainage. The objections to drainage are the time required for cure, and the possibility of a permanent fistula remaining. In order to shorten time, Pearce Gould and Fenger made their incisions posteriorly below the twelfth rib, as in lumbar nephrectomy, and obtained cure very quickly.

[1] New York Med. Rec., Dec. 22, 1894. [2] Lancet, Sept. 15, 1894. [3] Brit. Med. Journ., Nov. 10, 1894, and Il Policlin, Aug. 15, 1894. [4] Annals of Surgery, Dec., 1894. [5] Brit. Med. Journ., Nov. 3, 1894. [6] Med. Week, Sept. 14, 1894. [7] Annals of Surgery, Oct., 1894. [8] Med. Week, Sept. 14, 1894. [9] Med. Chronicle, Sept., 1894, and Beit. z. Centr. für Chir., No. 30, 1894. [10] Med. Chron., Nov., 1894, and Centr. für Gyuäkol, No. 27.

